# Characteristics and Treatment Outcomes of Out-of-Hospital Cardiac Arrests Occurring in Public Places: A National Population-Based Observational Study

**DOI:** 10.3390/jpm13081191

**Published:** 2023-07-26

**Authors:** Young Taeck Oh, Chiwon Ahn

**Affiliations:** 1 Department of Emergency Medicine, Hallym University Dongtan Sacred Heart Hospital, Hwaseong 18450, Republic of Korea; powerfreeze@hanmail.net; 2 Department of Emergency Medicine, College of Medicine, Chung-Ang University, Seoul 06974, Republic of Korea

**Keywords:** bystander intervention, out-of-hospital cardiac arrest, public places, South Korea

## Abstract

Sudden cardiac arrest, particularly out-of-hospital cardiac arrest (OHCA), is a global public health concern. However, limited research exists on the epidemiology of OHCAs occurring in public places, trends and impact of bystander intervention, and influence of extraordinary circumstances. This study investigated the epidemiological factors, bystander characteristics, and outcomes of OHCAs that occurred in public places in South Korea from 2016 to 2021 and analyzed the impact of the coronavirus disease 2019 (COVID-19) pandemic. A retrospective analysis was conducted using an Out-of-Hospital Cardiac Arrest Surveillance database, including 33,206 cases of OHCA that occurred in public places. Cases with do-not-resuscitate orders or insufficient data were excluded. A steady increase in bystander-performed cardiopulmonary resuscitation over the years and a constant decrease in bystander automated external defibrillator (AED) use were observed. Survival-to-discharge rates for OHCAs remained relatively steady until a marginal decrease was observed during the pandemic (pandemic, 13.1%; pre-pandemic, 14.4%). Factors affecting survival included the presence of a shockable rhythm, witnessed arrest, cardiac arrest due to disease, use of bystander AED, and period relative to the COVID-19 pandemic. These findings emphasize the critical role of bystanders in outcomes of OHCAs and inform public health strategies on better management of OHCAs in public places.

## 1. Introduction

Sudden cardiac arrest is a major global public health concern [1]. More than 30,000 victims occur annually in out-of-hospital settings in South Korea; however, survival remains low [2]. Out-of-hospital cardiac arrests (OHCAs) occurring in public spaces constitute a distinct subset of cardiac emergencies, distinguished by unique epidemiological characteristics and challenges [3,4,5,6]. As dynamic social spaces, such as streets, parks, shopping centers, and transportation hubs, public places engage a diverse population in various activities at any given moment. OHCAs occurring in public places may manifest distinctive patterns regarding age groups, prevalence of certain risk factors, time of occurrence, and causative factors compared to those occurring in residential or healthcare settings [6,7]. A detailed understanding of these differences could be vital in shaping the management of OHCAs occurring in public places, including improving response time, identifying high-risk locations, and enhancing the accessibility and utilization of emergency medical services.

With increasing public awareness and intervention during cardiac emergencies, bystanders have become critical factors determining outcomes of OHCAs occurring in public spaces [7,8]. Bystanders can potentially transform the survival rates through immediate recognition, initiation of cardiopulmonary resuscitation (CPR), and application of automated external defibrillators (AEDs) [8,9,10]. However, the trend, extent, and efficacy of bystander interventions have been variable and are likely influenced by factors such as public education, availability of AEDs, and sociocultural dynamics [11]. Additionally, the coronavirus disease 2019 (COVID-19) pandemic added another layer of complexity to the equation [12,13,14]. Pandemic-induced changes in public behavior, restrictions, and fear of infection may have influenced bystander intervention rates and, subsequently, the outcomes of OHCAs [12].

Despite these crucial issues, there is a conspicuous gap in the existing literature regarding the epidemiology of OHCAs occurring in public places, the trends and impact of bystander intervention, and the influence of extraordinary circumstances, such as the COVID-19 pandemic. Therefore, this study aimed to investigate the pre-hospital and epidemiological factors and outcomes of OHCAs that occurred in public places in South Korea between 2016 and 2021. We noted the findings in light of the pandemic and conducted further investigations. Such an understanding is essential for shaping better public health strategies, policy planning, and improving overall survival outcomes of OHCAs.

## 2. Materials and Methods

### 2.1. Study Design, Setting, and Data Source

This retrospective observational study used the nationwide, population-based Out-of-Hospital Cardiac Arrest Surveillance (OHCAS) database (managed by the Korea Disease Control and Prevention Agency (KDCA) (https://www.kdca.go.kr/, accessed on 3 July 2023) to evaluate the characteristics of OHCAs occurring in public places and the prognostic factors associated with the survival and good neurological outcomes from January 2016 to December 2021. The database contains information on all patients presenting with acute cardiac arrest transported to medical facilities by the emergency medical service (EMS), amounting to approximately 30,000 patients each year. As the study data were anonymous, the KDCA has allowed research using this database to be conducted, and this work was exempt from assessment by the institutional review board.

In South Korea, government-owned public EMS is available 24 h a day, 365 days a year, and is administered by 19 fire station headquarters under the National Fire Agency [15]. An ambulance is dispatched to the location of the OHCA patient in response to a call, and the patient is transported to a hospital. Before arriving at a hospital, paramedics perform CPR using an AED. Under the supervision of a physician, CPR can be suspended, or advanced airway techniques can be administered; however, drugs for advanced cardiac life support cannot be administered [16]. Information is transmitted from paramedics to the hospital upon the transfer of the patient. Resuscitation treatments at the hospital and after return of spontaneous circulation (ROSC) are administered according to each hospital’s protocol.

The OHCAS database uses patient data extracted from the EMS data registry and hospital medical records. Medical record investigators from the KCDA visit medical facilities to investigate the medical records of OHCA patients in relation to treatments and outcomes and to verify compliance with the Utstein style [17] and the Resuscitation Outcomes Consortium Project [18]. The database uses a customized survey form to record the information of individuals and settings; emergency medical services; emergency department care; hospital procedures; and outcomes at discharge, including survival and neurological outcomes.

### 2.2. Study Population and Classification of Arrest Location

In this study, we included only OHCAs that occurred in public places. Patients with do-not-resuscitate orders, traumatic cardiac arrest, invalid pre-hospital data, and unknown final outcomes were excluded from the study.

The OHCAS database contains arrest location information, which is divided into seven subcategories under a broad classification of public places: (1) roads/highways, (2) public buildings, (3) leisure facilities, (4) industrial facilities, (5) commercial facilities, (6) terminals, and (7) other public places. Detailed locations corresponding to each domain of the subclass were predetermined, provided via the OHCAS database manual, and are shown in Supplementary Table S1.

### 2.3. Variables

Data on several variables were collected, including age, sex, place of arrest, witnessed arrest, bystander CPR performed, rhythms initially monitored during the pre-hospital interval (non-shockable vs. shockable), and pre-hospital and in-hospital ROSC. A shockable rhythm was defined as the initial rhythm of pulseless ventricular tachycardia or fibrillation.

The OHCAS data collection period included the COVID-19 pandemic period, during which policies, such as social distancing, self-isolation, and restrictions on outdoor activities, were implemented [19]. As these could affect variables in the pre-hospital and hospital stages of OHCAs that occur in public places, we included a variable encoding the period of occurrence of OHCAs relative to the COVID-19 pandemic, defined based on the date of the World Health Organization’s pandemic declaration, 11 March 2020.

### 2.4. Outcome Measures

The primary outcome of this study was survival to discharge, which was characterized by the normal discharge or transfer of the patient to another healthcare facility for ongoing care following acute treatment. A favorable neurological outcome was the secondary outcome. The Cerebral Performance Category (CPC) score was used to classify neurological results. CPC scores of 1 and 2 were indicative of good neurological outcomes.

### 2.5. Statistical Analyses

The data were analyzed using Excel 2019 (Microsoft, Redmond, WA, USA) and R (version 4.3.1, The R Foundation for Statistical Computing, Vienna, Austria). Descriptive statistics were used to describe the baseline characteristics. For continuous variables, values are presented as the means ± standard deviations. Normally distributed variables were analyzed using Student’s *t*-test between groups. Categorical variables are expressed as frequencies and percentages. The χ^2^ test or Fisher’s exact test was used to analyze categorical variables using contingency tables. To identify the outcome predictors, covariates, including the binary variable of the period relative to the COVID-19 pandemic (pandemic or pre-pandemic), were evaluated using multivariate analysis. Logistic regression using the “enter” method was independently performed, adjusting for sex, age, witnessed arrest, bystander CPR, AED use, shockable rhythm, cause of arrest, and the period relative to the COVID-19 pandemic. *p* < 0.05 was considered statistically significant.

## 3. Results

**Figure 1:**
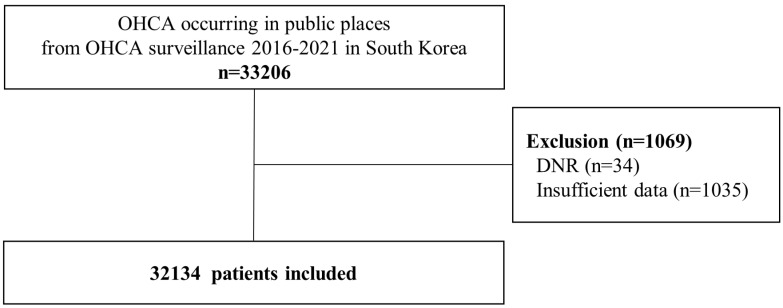
Flow diagram depicting the enrollment of the study population. DNR, do-not-resuscitate; OHCA, out-of-hospital cardiac arrest.

We identified 33,206 patients who suffered OHCAs in public places between January 2016 and December 2021. We excluded patients who had do-not-resuscitate orders (*n* = 34) and those with insufficient data (*n* = 1035). After the exclusion of 1069 patients, 32,134 patients who suffered OHCAs in public places were finally included in the study (Figure 1).

**Figure 2:**
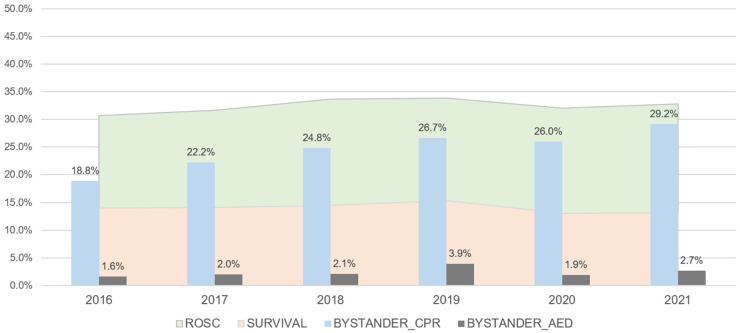
Graph showing the trends of bystander intervention and clinical outcomes of out-of-hospital cardiac arrests that occurred in public places from 2016 to 2021.

The proportion of males among the OHCA patients was 78.1% in 2016 and 79.6% in 2021. The rate of witnessed arrest rates were 58.3% and 57.7% in 2016 and 2021, respectively. The rate of bystander CPR implementation for OHCAs occurring in public places gradually increased (18.8, 22.2, 24.8, 26.7, 26.0, and 29.2% from 2016 to 2021). Meanwhile, the rate of bystander AED use remained similar from 2016 to 2021 (1.6, 2.0, 2.1, 3.9, 1.9, and 2.7% from 2016 to 2021). The rate of survival to discharge of patients with OHCA in public places was similar between 2016 and 2019, with a marginal decrease in 2020 and 2021 (14.0, 14.0, 14.5, 15.4, 13.0, and 13.1% from 2016 to 2021) (Table 1 and Figure 2).

Seven subcategories of public places were analyzed in detail. Roads or highways and commercial facilities were the most common places of occurrence of OHCAs (39.4% and 26.4%, respectively). The occurrence of OHCAs caused by disease was low on roads or highways and at industrial facilities (17.1% and 38.8%, respectively) and was >75% in other places. The rate of bystander AED use was 10.6% at terminals and only 0.3% on roads or highways. In addition, the rate of bystander AED use in commercial facilities was low (1.8%; Table 2).

We compared the COVID-19 pandemic and pre-pandemic periods for OHCAs that occurred in public places. The rate of implementation of bystander CPR during the pandemic was significantly higher than that during the pre-pandemic period (27.3% and 22.2%, respectively; *p* < 0.001). The rate of OHCAs caused by disease during the pandemic was higher than that during the pre-pandemic period (55.7% and 52.2%, respectively; *p* < 0.001). Survival to discharge and favorable neurological outcomes significantly decreased during the pandemic (13.1%, 14.4%, *p* = 0.002 and 7.7%, 8.2%, *p* < 0.001, respectively) (Table 3).

**Figure 3:**
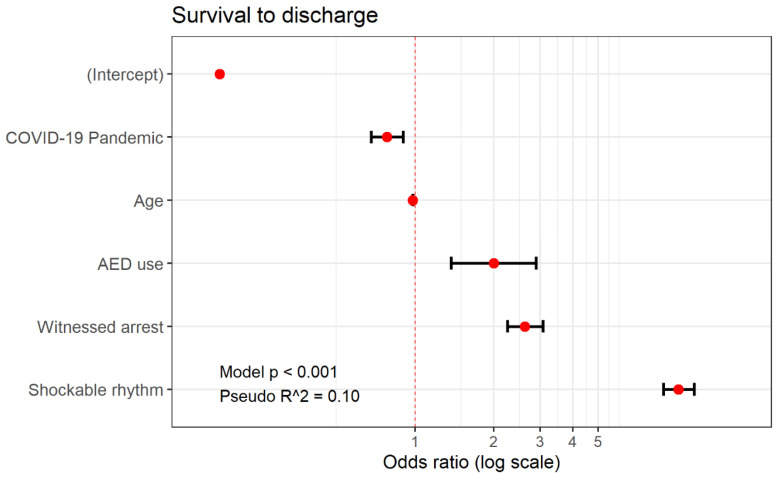
Forest plot of adjusted odds ratios and 95% confidence intervals for survival to discharge of patients who suffered from out-of-hospital cardiac arrests in public places.

Regarding the factors that affected the survival to discharge of patients who suffered OHCAs in public places, shockable rhythm was the most influential variable after adjusting for variables (odds ratio (OR), 10.12; 95% confidence interval (CI), 8.85–11.59, *p* < 0.001), followed by cardiac arrest due to disease and use of bystander AED (OR, 2.00; 95% CI, 1.37–2.90, *p* < 0.001). The period to the COVID-19 pandemic was associated with low survival to discharge (OR, 0.78; 95% CI, 0.68–0.90, *p* < 0.001) (Table 4 and Figure 3).

## 4. Discussion

This study provided a comprehensive epidemiological assessment of OHCAs that occurred in public places in South Korea from 2016 to 2021, exploring the factors associated with outcomes and analyzing the impact of bystander interventions. The findings highlighted the crucial role of bystanders in OHCA management, with the rate of bystander-performed CPR steadily increasing from 18.8% in 2016 to 29.2% by 2021. These figures highlighted the growing public awareness and intervention in cardiac emergencies. However, bystander use of AED remained low during the study period, indicating it as an area that requires further public health interventions. Upon the examination of the survival-to-discharge rates of patients who suffered OHCAs in public places, our data presented a marginal dip during the last 2 years of the study, which coincided with the COVID-19 pandemic period.

Previous studies have shown that OHCAs occurring in public places are frequently witnessed and have a high chance of bystander intervention [7,8]. In contrast to OHCA occurrences in residences, those occurring in public places involve younger people with fewer comorbidities [6,20,21]. However, this is not the only manner of describing public places. For example, a study showed that individuals who suffer OHCAs in workplaces have higher survival rates [22]. Additional research revealed that cardiac arrests occurring at terminals are frequently witnessed and such individuals receive more bystander intervention [23]. Consequently, it is important to distinguish between differences based on specific categories, even among public places.

Compared to other public places in this study, OHCAs that occurred on roads and highways showed distinctive characteristics. Trauma and other non-disease-related causes of OHCAs were common, whereas bystander intervention was uncommon (CPR, 11.6%; AED, 0.3%); the survival-to-discharge rate was only 7.0%. In contrast, leisure facilities, public buildings, and terminals showed considerable levels of bystander intervention (CPR: 41.5%, 40.1%, and 39.4%; AED use: 6.2%, 7.1%, and 10.6%, respectively). There is an apparent association between effective basic life support training and the presence of competent bystanders in public buildings, which typically includes schools [9,24,25]. AEDs are appropriately positioned in these buildings, and their relative accessibility increases the likelihood of immediate use [26]. The presence of multiple AEDs in transport terminals or transit stations contributes to a high rate of bystander AED use [27,28,29]. Terminals where large groups of people gather have numerous AEDs installed, which increase the likelihood of bystanders use. This study demonstrated that the use of AEDs at terminals was relatively high (10.6%). Public buildings, leisure facilities, and major locations, such as terminals, are required by law to install and administer AEDs. South Korea regulates the installation and maintenance of AEDs according to these laws. The frequency of AED use demonstrated in this study conformed to observations regarding regulations on AED installation.

The overall low implementation and usage rate of AEDs in various public places are problems requiring improvement. The rate of AED use for OHCAs occurring in public places is between 1.0% and 12.8% in other countries; however, it remains extremely low worldwide [7,30,31,32,33,34]. Kitamura et al. reported that the continuous deployment of Public Access Defibrillation and the increase in AED purchases and placements led to a 9-year increase in the use of bystander AEDs from 1.1% to 16.5% in Japan [35]. Despite legislation mandating the installation and administration of AEDs in South Korea, the considerable disparities in AED utilization rates across public spaces may be attributed to limited AED accessibility or a lack of AED use awareness in certain areas. Kwon et al. noted that the placement of AEDs mandated by law can impede placement efficiency if accessibility is not considered. In addition, whereas training on AED use was included, it did not cover how to locate and retrieve an AED [36]. Even if individuals are technically capable of using an AED, it may be difficult to increase their usage if individuals are unaware of how to access the device. To increase bystander use of AEDs in public places for OHCAs, it is necessary to consider these points.

The pandemic substantially impacted OHCA characteristics. Kim et al. reported that the pandemic led to an increase in OHCAs at home, a decrease in AED use, and an increase in EMS response time compared to the pre-pandemic period [12]. This alteration in epidemiological characteristics is associated with a decline in survival rates and favorable neurological outcomes of OHCA patients [12,37]. Considerable changes in emergency medical systems that influence these outcomes include the concentration of medical resources for COVID-19 treatment, emergence of a relative gap in critically ill patients, effects of social distancing measures, self-isolation, and diminished social activities [37]. In this study, when comparing the characteristics of OHCAs that occurred in public places during the pandemic and pre-pandemic periods, differences were found. Notably, there were more instances of bystander CPR implementation during the pandemic. Ball et al. also reported an increase in bystander CPR implementation during the pandemic, which was attributed to an increase in occurrence of OHCAs in residential areas [38]. In addition, meta-analyses found no significant difference between the CPR administered by bystanders before and during the pandemic [12,37]. This study attributed the increase in bystander CPR implementation during the pandemic in public places in South Korea to continued training on remote cardiopulmonary resuscitation, increased public awareness of CPR, and the easing of COVID-19 fears after the initial phase of the pandemic, owing to the availability of medicines and vaccines. The 29.2% rate of bystander CPR conducted in 2021 reflects this finding. This trend needs to be compared to the rate of bystander CPR implementation in public places in the following year.

The clinical outcomes of OHCAs that occurred in public places was markedly affected by factors such as the use of a bystander AED and the period relative to the COVID-19 pandemic, with a shockable rhythm having a substantial impact on the survival rate, which is consistent with prior knowledge. Research suggests that cardiac arrests in public places may be more likely to arise from certain heart conditions that result in shockable rhythms, in contrast to those occurring at home, which may often be due to medical conditions leading to non-shockable rhythms [6,7]. As cardiac arrests in public places are more likely to be witnessed, interventions, such as immediate CPR or defibrillation, can commence sooner. Consequently, shockable rhythms tend to have a more significant influence on outcomes than non-shockable rhythms.

This study has some limitations. First, despite using a nationwide dataset, the study utilized data collected by healthcare providers in the field, which may have resulted in a high probability of missing data and bias. Second, combining distinct public places into one large category for analysis can reduce representative homogeneity. For example, convenience stores, which are commercial facilities interspersed with residential areas and operate 24 h a day, may exhibit characteristics distinct from those of other commercial facilities. However, interpreting them as a single category may not adequately reflect these distinctions. Third, the study did not accurately reflect hospital treatment processes for each individual. Owing to lack of access to this information, we were unable to analyze the effect of drug use and hospital events on treatment outcomes. Fourth, although the purpose of this research was to report the characteristics of South Korean data, it is difficult to generalize these findings. The application of South Korea’s emergency medical systems, the function and capabilities of paramedics, and urban characteristics in other countries can be complicated by differences in these aspects. Finally, because this was a retrospective study, there is a substantial possibility of selection bias and the occurrence of latent confounders, which could limit the findings.

## 5. Conclusions

This study investigated the recent occurrence of OHCAs in public places in South Korea, offering insights into OHCAs occurring in these locations. The findings revealed a rising rate of bystander CPR implementation; however, AED use by bystanders remained low. Therefore, enhancing AED accessibility, boosting awareness, and adapting emergency systems are essential measures that need to be taken for improving outcomes for individuals who suffer OHCAs in public spaces.

## Figures and Tables

**Table 1:** Baseline characteristics for out-of-hospital cardiac arrests that occurred in public places between 2016 and 2021.

Variable	2016 (*N* = 5410)	2017 (*N* = 5539)	2018 (*N* = 5587)	2019 (*N* = 5379)	2020 (*N* = 5178)	2021 (*N* = 5044)	*p* Value
Sex, Male	4225 (78.1%)	4359 (78.7%)	4321 (77.3%)	4206 (78.2%)	4117 (79.5%)	4017 (79.6%)	0.030
Age	57.7 ± 17.7	58.8 ± 17.5	59.2 ± 17.5	60.2 ± 17.2	60.1 ± 17.2	60.1 ± 17.5	<0.001
Witnessed arrest	2654 (58.3%)	3185 (63.8%)	2769 (56.6%)	2327 (53.2%)	2605 (56.1%)	2733 (57.7%)	<0.001
Bystander CPR	996 (18.8%)	1189 (22.2%)	1332 (24.8%)	1358 (26.7%)	1302 (26.0%)	1431 (29.2%)	<0.001
Bystander AED use	16 (1.6%)	24 (2.0%)	28 (2.1%)	53 (3.9%)	25 (1.9%)	39 (2.7%)	0.002
Cause, disease	2636 (48.7%)	2818 (50.9%)	2857 (51.1%)	2984 (55.5%)	2851 (55.1%)	2812 (55.7%)	<0.001
Cause, cardiac origin	2498 (46.2%)	2705 (48.8%)	2686 (48.1%)	2848 (52.9%)	2741 (52.9%)	2673 (53.0%)	0.001
Shockable rhythm	1065 (20.6%)	1056 (19.7%)	1079 (20.0%)	1099 (20.8%)	1025 (20.3%)	949 (19.1%)	0.260
Pre-hospital ROSC	508 (9.4%)	574 (10.4%)	585 (10.5%)	571 (10.6%)	499 (9.6%)	497 (9.9%)	0.194
Underlying disease							
Hypertension	795 (14.7%)	860 (15.5%)	938 (16.8%)	967 (18.0%)	896 (17.3%)	1002 (19.9%)	<0.001
Diabetes mellitus	493 (9.1%)	564 (10.2%)	575 (10.3%)	599 (11.1%)	582 (11.2%)	639 (12.7%)	<0.001
Heart disease	423 (7.8%)	488 (8.8%)	521 (9.3%)	564 (10.5%)	489 (9.4%)	512 (10.2%)	<0.001
Renal disease	85 (1.6%)	86 (1.6%)	77 (1.4%)	110 (2.0%)	113 (2.2%)	134 (2.7%)	<0.001
Respiratory disease	88 (1.6%)	109 (2.0%)	112 (2.0%)	125 (2.3%)	108 (2.1%)	138 (2.7%)	0.003
Stroke	117 (2.2%)	145 (2.6%)	156 (2.8%)	176 (3.3%)	159 (3.1%)	169 (3.4%)	0.003
Hyperlipidemia	60 (1.1%)	93 (1.7%)	104 (1.9%)	128 (2.4%)	128 (2.5%)	165 (3.3%)	<0.001
PCI	180 (3.3%)	229 (4.1%)	278 (5.0%)	270 (5.0%)	241 (4.7%)	271 (5.4%)	<0.001
TTM	113 (2.1%)	159 (2.9%)	220 (3.9%)	200 (3.7%)	178 (3.4%)	179 (3.5%)	<0.001
Mechanical CPR	163 (3.0%)	250 (4.5%)	411 (7.4%)	429 (8.0%)	734 (14.2%)	920 (18.2%)	<0.001
ECMO CPR	36 (0.7%)	76 (1.4%)	81 (1.4%)	73 (1.4%)	71 (1.4%)	76 (1.5%)	0.001
ROSC	1662 (30.7%)	1754 (31.7%)	1883 (33.7%)	1821 (33.9%)	1660 (32.1%)	1653 (32.8%)	0.003
Survival to discharge	755 (14.0%)	778 (14.0%)	811 (14.5%)	826 (15.4%)	675 (13.0%)	663 (13.1%)	0.006
Favorable neurological outcome *	365 (6.7%)	482 (8.7%)	473 (8.5%)	483 (9.0%)	382 (7.4%)	401 (8.0%)	<0.001

AED, automated external defibrillator; CPR, cardiopulmonary resuscitation; ECMO, extracorporeal membrane oxygenation; PCI, percutaneous coronary intervention; ROSC, return of spontaneous circulation; TTM, target temperature management. * Favorable neurological outcomes included cerebral performance categories 1 and 2.

**Table 2:** Outcomes of out-of-hospital cardiac arrests that occurred in public places, according to the detailed classification of public places.

Variable	Roads or Highways (*N* = 12,673)	Public Buildings (*N* = 741)	Leisure Facilities (*N* = 1086)	Industrial Facilities (*N* = 3544)	Commercial Facilities (*N* = 8496)	Terminals (*N* = 837)	Other Public Places (*N* = 4760)	*p* Value
Sex, male	9471 (74.7%)	564 (76.1%)	872 (80.3%)	3420 (96.5%)	6879 (81.0%)	702 (83.9%)	3337 (70.1%)	<0.001
Age	56.6 ± 18.8	57.3 ± 21.1	58.7 ± 17.2	53.9 ± 11.6	62.0 ± 16.5	63.5 ± 15.3	65.6 ± 16.2	<0.001
Witnessed arrest	6768 (70.7%)	474 (65.7%)	616 (57.9%)	1979 (61.2%)	3700 (45.0%)	469 (58.6%)	2267 (49.5%)	<0.001
Bystander CPR	1465 (11.6%)	297 (40.1%)	451 (41.5%)	890 (25.1%)	2892 (34.0%)	330 (39.4%)	1283 (27.0%)	<0.001
Bystander AED use	5 (0.3%)	21 (7.1%)	28 (6.2%)	20 (2.2%)	53 (1.8%)	35 (10.6%)	23 (1.8%)	<0.001
Cause, disease	2152 (17.0%)	593 (80.0%)	813 (74.9%)	1362 (38.4%)	7298 (85.9%)	701 (83.8%)	4039 (84.9%)	<0.001
Cause, cardiac origin	2053 (16.2%)	562 (75.8%)	787 (72.5%)	1302 (36.7%)	6912 (81.4%)	671 (80.2%)	3864 (81.2%)	0.070
Shockable rhythm	1073 (8.9%)	298 (40.8%)	450 (42.5%)	793 (23.3%)	1984 (23.6%)	277 (33.5%)	1398 (29.7%)	<0.001
Pre-hospital ROCS	490 (3.9%)	183 (24.7%)	299 (27.5%)	362 (10.2%)	1106 (13.0%)	148 (17.7%)	646 (13.6%)	<0.001
ROSC	2607 (20.6%)	355 (47.9%)	558 (51.4%)	1005 (28.4%)	3433 (40.4%)	370 (44.2%)	2105 (44.2%)	<0.001
Survival to discharge	890 (7.0%)	225 (30.4%)	356 (32.8%)	506 (14.3%)	1503 (17.7%)	168 (20.1%)	860 (18.1%)	<0.001
Favorable neurological outcome *	336 (1.6%)	157 (13.9%)	276 (16.9%)	296 (5.5%)	897 (6.7%)	119 (9.2%)	505 (6.5%)	<0.001

AED, automated external defibrillator; CPR, cardiopulmonary resuscitation; ROSC, return of spontaneous circulation. * Favorable neurological outcomes included cerebral performance categories 1 and 2.

**Table 3:** Outcomes of out-of-hospital cardiac arrests that occurred in public places in the pandemic and pre-pandemic periods.

Variable	Pre-Pandemic (*N* = 22,897)	Pandemic (*N* = 9240)	*p* Value
Sex, male	5024 (21.9%)	1868 (20.2%)	0.001
Age	59.1 ± 17.5	60.0 ± 17.3	<0.001
Witnessed arrest	11,410 (58.0%)	4863 (57.1%)	0.170
Bystander CPR	5090 (22.2%)	2518 (27.3%)	<0.001
Bystander AED use	124 (2.4%)	61 (2.4%)	1.000
Cause, disease	11864 (51.8%)	5094 (55.1%)	<0.001
Cause, cardiac origin	11287 (49.3%)	4864 (52.6%)	0.349
Shockable rhythm	4493 (20.3%)	1780 (19.6%)	0.212
Prehospital ROCS	2328 (10.2%)	906 (9.8%)	0.339
ROSC	7442 (32.5%)	2991 (32.4%)	0.829
Survival to discharge	3300 (14.4%)	1208 (13.1%)	0.002
Favorable neurological outcome	1874 (8.2%)	712 (7.7%)	<0.001

AED, automated external defibrillator; CPR, cardiopulmonary resuscitation; ROSC, return of spontaneous circulation.

**Table 4:** Univariate and multivariate logistic regression analysis of survival to discharge of patients who suffered from out-of-hospital cardiac arrests in public places.

Factor	Univariate OR (95% CI)	*p* Value	Adjusted OR	*p* Value
Sex, male	0.84 (0.77–0.91)	<0.001	-	
Age	0.99 (0.99–0.99)	<0.001	0.98 (0.98–0.98) *	<0.001
Witnessed arrest	2.70 (2.50–2.91)	<0.001	2.62 (2.25–3.07) *	<0.001
Bystander CPR	1.05 (1.03–1.07)	<0.001	-	
Bystander AED use	2.24 (1.66–3.01)	<0.001	2.00 (1.37–2.90) *	<0.001
Shockable rhythm	10.79 (10.06–11.57)	<0.001	10.12 (8.85–11.59) *	<0.001
Cause of arrest, disease	4.76 (4.40–5.16)	<0.001	2.11 (1.87–2.38) *	<0.001
Period relative to the pandemic	0.89 (0.83–0.96)	0.002	0.78 (0.68–0.90) *	<0.001

Adjusted for sex, age, witnessed arrest, bystander CPR, AED use, shockable rhythm, cause of arrest, and the period relative to the COVID-19 pandemic. * Factors included in the final logistic regression model for survival to discharge. AED, automated external defibrillator; CI, confidence interval; COVID-19, coronavirus disease 2019; CPR, cardiopulmonary resuscitation; OR, odds ratio.

## Data Availability

The datasets generated during the current study are available from the corresponding author upon reasonable request.

## Supplementary Materials

The following supporting information can be downloaded at: https://www.mdpi.com/article/10.3390/jpm13081191/s1, Table S1: Detailed classification of public places by category.
