# Supplementary material for: Characteristics and Treatment Outcomes of Out-of-Hospital Cardiac Arrests Occurring in Public Places: A National Population-Based Observational Study
Source: J Pers Med. 2023 Jul 26;13(8):1191. doi: 10.3390/jpm13081191 (PMC10455591; doi:10.3390/jpm13081191)
Supplement: Supplementary file 1 [file jpm-13-01191-s001.zip › jpm-2533025-supplementary.pdf]

Supplementary Table S1. Detailed classification of public places by category

Classification	Details
Roads/Highways	Roads, car roads, paved roads, highways, intersections, inside a car driving on a road, etc.
Public buildings	Schools, public playgrounds, courts, police stations, community centers, libraries, public welfare centers, etc.
Leisure facilities	Amusement parks, botanical gardens and plazas, parks, opera houses, cinemas, theaters, museums, galleries, recreational areas along the Han River/reservoirs, etc.
Industrial facilities	Gas plants, all buildings under construction (including houses under construction), tunnels under construction, industrial complexes, factory buildings, factory premises (including internal roads, parking lots, and warehouses), mines, dry docks, power plants, docks, coastguard stations, and other coastal facilities, workshops, shipyards, quarries, railway maintenance yards, etc.
Commercial facilities	Offices, saunas, bathhouses, shopping malls, supermarkets, convenience stores, markets, banks, gas stations, bars, cafes, hotels, restaurants, rest areas, wedding halls, funeral homes, etc.
Terminals	Railway stations, bus stops, airports, subways, piers, ports, docked ships.
Other public places	Sidewalks, pedestrian bridges, vehicle checkpoints, inside trains, churches, cathedrals, temples, public parking lots, prayer halls, cruise ships, passenger ships, airplanes, welfare centers, community centers, village halls, nursing homes, senior health centers, kindergartens, daycare centers, etc.
